# The potential of carbon-based nanomaterials in hepatitis C virus treatment: a review of carbon nanotubes, dendrimers and fullerenes

**DOI:** 10.1186/s11671-023-03895-5

**Published:** 2023-09-16

**Authors:** Karim Nader, Amro Shetta, Sameh Saber, Wael Mamdouh

**Affiliations:** 1 Department of Mechanical Engineering, School of Sciences and Engineering, The American University in Cairo (AUC), Cairo, 11835 Egypt; 2 Department of Chemistry, School of Sciences and Engineering, The American University in Cairo (AUC), Cairo, 11835 Egypt; 3 Department of Pharmacology, Faculty of Pharmacy, Delta University for Science and Technology, Gamasa, 11152 Egypt

**Keywords:** HCV, Dendrimers, Fullerenes, CNTs

## Abstract

HCV, hepatitis C virus, is a virus that causes damage to the liver. Both chronic infection or lack of treatment increase morbidity except if it is an acute infection, as the body clears the virus without any intervention. Also, the virus has many genotypes, and until now, there has yet to be a single treatment capable of affecting and treating all these genotypes at once. This review will discuss the main and most used old treatments, IFN-a, PEG IFN-a, Ribavirin, Celgosvir, and sofosbuvir alone and with the combination of other drugs and their drawbacks. They should be given in combination to improve the effect on the virus compared with being administrated independently, as in the case of sofosbuvir. For these reasons, the need for new treatments and diagnostic tools arises, and the rule of nanotechnology comes here. The role of carbon nanotubes, dendrimers, and fullerenes will be discussed. CNTs, carbon nanotubes, are one-dimensional structures composed of a cylindrical sheet of graphite and are mainly used for diagnostic purposes against HCV. Dendrimers, three-dimensional highly branched structures, are macromolecules that provide better drug delivery and treatment options due to their unique structure that can be modified, producing versatile types; each has unique properties. Fullerenes which are cage like structures derived and closely related to CNTs, and composed of carbon atoms that can be substituted by other atoms which in return open unlimited usage for these carbon based materials. Fullerenes rule is unique since it has two mechanisms that prevent the virus from binding and acting on the virus-replicating enzyme. However, their charge needs to be determined; otherwise, it will lead to cytotoxicity. Lastly, no review has been done on the role of nanotechnology against HCV yet.

## Hepatitis C viral infection

Hepatitis C virus (HCV), an RNA virus with a single-stranded genome, has raised global concerns due to its significant impact on morbidity and mortality, often necessitating liver transplantation and resulting in liver-related fatalities worldwide. Structurally, the virus possesses a lipid envelope composed of LDL and VLDL, imparting a distinct dark appearance on its surface. Notably, the outer surface of the virion features 6nm projections formed by glycoproteins E1 and E2, which are embedded within the lipid bilayer. HCV primarily targets the liver, triggering inflammation, and can be transmitted through unsafe injection practices, contaminated needles and syringes, unscreened blood transfusions, vertical transmission from an infected mother to her infant, and, to a lesser extent, sexual intercourse involving exposure to infected blood, although such cases are relatively rare. It is important to note that while sexual transmission among men occurs, it is not widespread. As for symptoms, many patients with HCV infection are asymptomatic, but individuals with symptomatic infections may experience fatigue, fever, vomiting, nausea, abdominal discomfort, dark urine, loss of appetite, and jaundice. The inflammatory response induced by HCV infection can range from mild acute hepatitis to severe chronic hepatitis, ultimately leading to patient mortality [1].

Acute cases of HCV typically do not manifest symptoms and are generally not life-threatening, as most patients spontaneously clear the virus within six months without any interventions. Evidence indicates that the body can naturally eliminate the virus, which is reflected in the presence of jaundice, elevated alanine transaminase levels, and the hepatitis B virus surface antigen. However, in severe cases, chronic hepatitis can progress to cirrhosis or hepatocellular carcinoma, a form of liver cancer. One significant challenge with chronic HCV is the absence of symptoms or noticeable problems until the disease has advanced. Consequently, regular check-ups, blood tests, liver enzyme tests, or liver ultrasounds are crucial for early diagnosis. According to the World Health Organization (WHO), approximately 58 million individuals are affected by chronic HCV, with around 1.5 million new cases emerging yearly. A study by Roudot Thoraval et al. revealed that 1.7 million new infections were reported in 2015, with the highest incidence observed in the Eastern Mediterranean region, followed by the European region, while the Southeast Asia region demonstrated the lowest incidence [2].

According to the Centers for Disease Control and Prevention (CDC), cirrhosis occurs in approximately 5 to 25 cases per 100 patients, and approximately 5% of these cases progress to hepatocellular carcinoma. The World Health Organization (WHO) reports that HCV-related deaths reach nearly 290,000 annually worldwide. It is important to note that re-infection can occur, meaning that recovering from an HCV infection does not provide lifelong protection. Patients can be infected again by the same or different HCV genotypes. HCV is classified into seven genotypes and 67 subtypes. Genotypes 2 and 3 account for approximately 30% of chronic HCV cases, with genotype 3 posing a higher risk of hepatic steatosis, fibrosis progression, and hepatocellular carcinoma compared to genotype 2. Geographically, the Eastern Mediterranean and European regions bear the highest disease burden, with approximately 12 million cases each, followed by Africa, with 9 million patients. The Americas have the lowest number of reported cases, totaling 5 million. Notably, 75% of these cases occur in low and middle-income countries. This study aims to highlight the current limitations in HCV treatment and explore the potential of nanotechnology, such as carbon nanotubes, dendrimers, and fullerenes, for effective drug delivery of antivirals targeting HCV and HIV. This innovative approach holds promise for treating infectious viruses [3].

CNTs, Dendrimers, and Fullerenes will be the scope that this review will focus on, highlighting their importance and uses against the hepatitis C virus, their challenges that need to be mitigated, and future recommendations. The literature will start with CNTs, which are mainly used as sensors, and these sensors are divided into sensors for HCV genotype detection and sensors used for anti-HCV drug concentration estimation (the majority of the sensors stand in this type). Then, dendrimers are mainly used as drug delivery systems; the main types mentioned are anionic and janus dendrimers. Lastly, fullerenes have been found to have a potent effect against HCV by blocking the enzymes bound to cell membrane replication and protease enzymes; however, their main issue is the low solubility and the higher production cost. This review is the only one of its type that has addressed the usage of carbon allotropes against HCV until now.

## Etiology of the disease

**Fig. 1:**
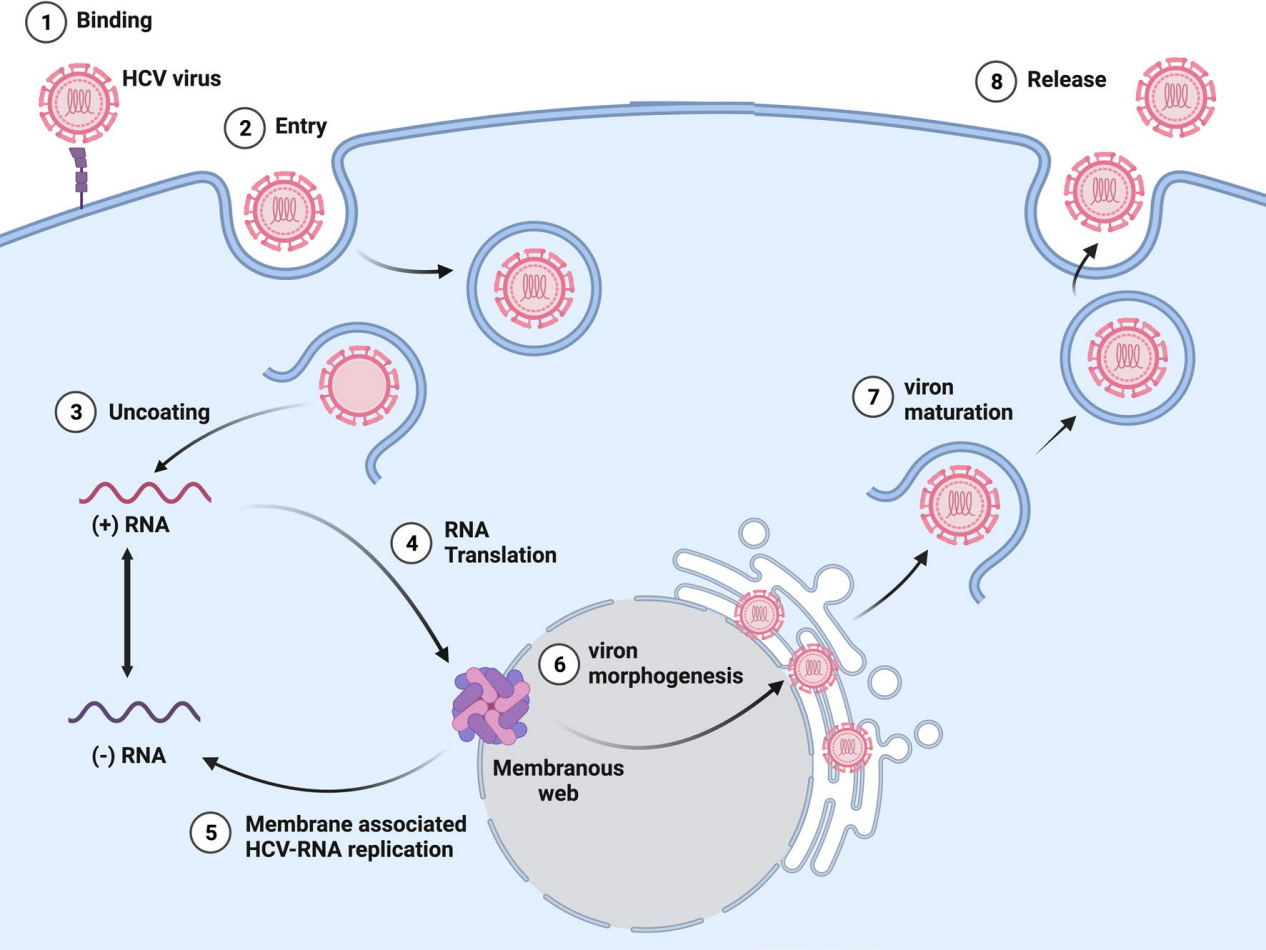
HCV infection mechanism. Figure is produced by BioRender.com (2023). Retrieved from https://app.biorender.com/biorender-templates [5]

HCV is a virus that is suspected to cause carcinoma either directly or indirectly. Compared with other viruses that cause carcinoma, HCV is less prominent. Regarding HCV mechanism of action represented in (Fig. 1), the virus positive RNA strand replicates out of the nucleus without interfering with the host’s genome. Instead, it interprets with the host’s molecular pathway that controls the cell cycle. To explain more, the main proteins of HCV, NS3, and NS5A, have been found to affect the function of tumor suppressor p53; however, the interaction of these proteins is not well understood yet. Another HCV protein is NS5B; this protein stimulates the PKC superfamily, and SOCS3 expression activates ERK/JNK cascades, activating STAT3 by phosphorylation. As a result, the activation of STAT3 evokes the expression of Bcl-2 and MMP-2, which eventually results in deregulation and apoptosis of the cell. Moving to indirect mechanisms, oxidative stress, chronic inflammation, and over-proliferation may increase the accumulation of mutations and oncogenic transformation [4].

## HCV treatments protocols and their drawbacks

As a matter of fact, the mentioned treatments aim to cure the HCV infection. Regrettably, there is currently no available vaccine for HCV that can prevent the negative outcomes caused by this virus right from the beginning.

### Interferon alfa (IFN-alfa)

In the early 1990s, interferon alfa (IFN-alfa) emerged as the first approved drug for chronic HCV and was initially administered as monotherapy for a 6-month duration. IFN exhibits potent antiviral activity by inhibiting viral replication through the induction of proteins encoded by IFN-stimulated genes. Additionally, it stimulates the production of IL-2 and IFN-gamma by promoting the differentiation of T-helper 1 cells, thereby favoring an antiviral immune response over Th2 cell-mediated immune responses. Furthermore, IFN possesses anti-inflammatory properties by modulating cytokines. Initially, the sustained virologic response (SVR) rates with IFN-alfa treatment ranged from 6 to 12%. However, extending the treatment duration to 12 months led to an improved SVR of 15% to 19%. Subsequently, adding ribavirin to IFN-alfa therapy increased SVR rates from 30 to 40%. More recently, the combination of ribavirin with pegylated IFN-alfa, which involves attaching polyethylene glycol to IFN-alfa to prolong its activity, significantly increased the SVR rates from 54 to 56% [6].

### Pegylated interferon (PEG IFN-a)

PEG IFN has superseded the use of IFN as a standalone treatment due to its superior efficacy. When used alone, PEG IFN achieves an impressive sustained virologic response (SVR) rate of 80%. However, when combined with ribavirin, the SVR rate further improves to 85%. According to Kamal et al., PEG IFN exhibits remarkable effectiveness against genotypes 2 and 3, with an SVR rate of 85%. However, its efficacy against genotypes 1 and 4 ranges between 40 and 50%. As a result, PEG IFN proves to be particularly effective in treating acute HCV cases, successfully resolving around half of chronic HCV cases8. It is important to note that PEG IFN treatment is associated with common side effects, including flu-like symptoms, psychiatric symptoms, fatigue, muscle pain, sleep disturbances, and hair loss [7].

### Sofosbuvir

Introducing another treatment option, Sofosbuvir, an inhibitor of the HCV NS5B polymerase, proves to be a significant breakthrough in managing chronic HCV cases. Recently approved by the FDA, it is often used with other therapies tailored to the specific infectious genotype of HCV. Combining sofosbuvir with ribavirin is particularly effective against genotypes 2 and 3. In contrast, the combination of sofosbuvir with ledipasvir is recommended for genotypes 1 and 4, as well as for patients co-infected with HCV and HIV or those who have previously failed a sofosbuvir/ribavirin treatment course.

Regarding the combination of sofosbuvir and ribavirin, an extensive clinical study conducted by Zeuzem et al. suggests that a 24-week therapy duration is more effective than a 12-week regimen. Notably, this combination regimen has demonstrated a high barrier to HCV resistance, with no detected instances of the S282T virus-resistant variant in any patient. However, it is essential to acknowledge that adverse effects have been observed with this combination therapy. Liver enzyme elevations, specifically alanine aminotransferase and aspartate aminotransferase, have been reported, leading to treatment discontinuation in some cases due to malaise and headache, particularly in the 12-week course. In the 24-week course, more frequent adverse effects include diarrhea and irritability; one patient even experienced suicidal ideation.

Overall, Sofosbuvir presents a promising treatment option for chronic HCV cases, and its combination with other agents offers improved efficacy. However, it is crucial to closely monitor patients for potential adverse effects and tailor the treatment duration to optimize outcomes and minimize complications.

### Sofosbuvir/daclatasvir

Daclatasvir (DCV) is a potent inhibitor targeting the N5SA replication enzyme, making it a valuable component in the treatment of HCV. This combination therapy is particularly beneficial for non-cirrhotic and cirrhotic patients and those who have undergone liver transplantation and are facing severe HCV recurrence. Additionally, it has proven to be effective against genotype 4 cases. The standard duration of the DCV-based regimen is 12 weeks, but it can be extended to 24 weeks for patients who have not achieved an effective cure with the sofosbuvir/ribavirin combination. Clinical studies conducted by Ossama et al. demonstrated an impressive sustained virologic response (SVR) rate of 95% in non-cirrhotic patients and 83% in cirrhotic patients. This makes DCV an essential treatment option for patients classified as "difficult to treat [8]."

The primary rationale behind utilizing DCV lies in its potent antiviral effects against genotypes 1 and 4, which are responsible for a significant proportion of HCV cases. Furthermore, DCV offers the advantages of reduced side effects and improved affordability compared to other treatments. The issue of major side effects can be effectively addressed by replacing less specific agents such as IFN and RBV with DCV. However, it is essential to acknowledge one of the limitations associated with DCV treatment, as highlighted by Lee. DCV exhibits a relatively low genetic barrier, necessitating its use in combination with another highly effective antiviral agent, such as sofosbuvir, to mitigate the risk of drug resistance.

In summary, DCV holds excellent promise as an integral component of HCV treatment regimens. Its efficacy, particularly in challenging cases, and its ability to minimize side effects and enhance affordability makes it a valuable therapeutic option. The potential drawback of a low genetic barrier can be effectively managed by combining DCV with other potent antiviral drugs, thereby maximizing treatment success rates [9]. Both sofosbuvir and DCV structures presented in (Table 1).

**Table 1:** Chemical structures for used drugs

Drug	Structure	References
Sofosbuvir		[10]
Daclatasvir (DCV)		[9]

## Nanotechnology

Nanotechnology, operating at the nanometer scale, has emerged as a promising multidisciplinary field for developing innovative solutions to combat side effects and revolutionize treatment approaches. Among these solutions, nanotechnology-based carriers have gained significant attention. Various types of carriers can be employed in antiviral drug delivery, including nanocapsules, liposomes, dendrimers, and fullerenes. Nanocapsules, characterized by their hollow sphere structure, offer a high drug load capacity within their cavity while maintaining low density. Liposomes, on the other hand, act as biodegradable and non-toxic carriers, often serving as nanovectors. With their spherical shape, liposomes possess an aqueous inner cavity surrounded by a phospholipid bilayer outer layer. This unique structure allows drug loading on the surface or within the inner cavity, enhancing bioavailability [11]. Silibinin, an active polyphenolic compound derived from milk thistle, has demonstrated potent inhibition of HCV entry and replication. However, its poor bioavailability poses a challenge. To overcome this limitation, liposomes have been utilized as carriers to increase the bioavailability of silibinin. In addition to liposomes, this review will explore the application of carbon nanotubes, dendrimers, and fullerenes in antiviral drug delivery, highlighting their potential in addressing drug delivery challenges and advancing HCV treatment strategies [12].

### Carbon nanotubes role against HCV

**Fig. 2:**
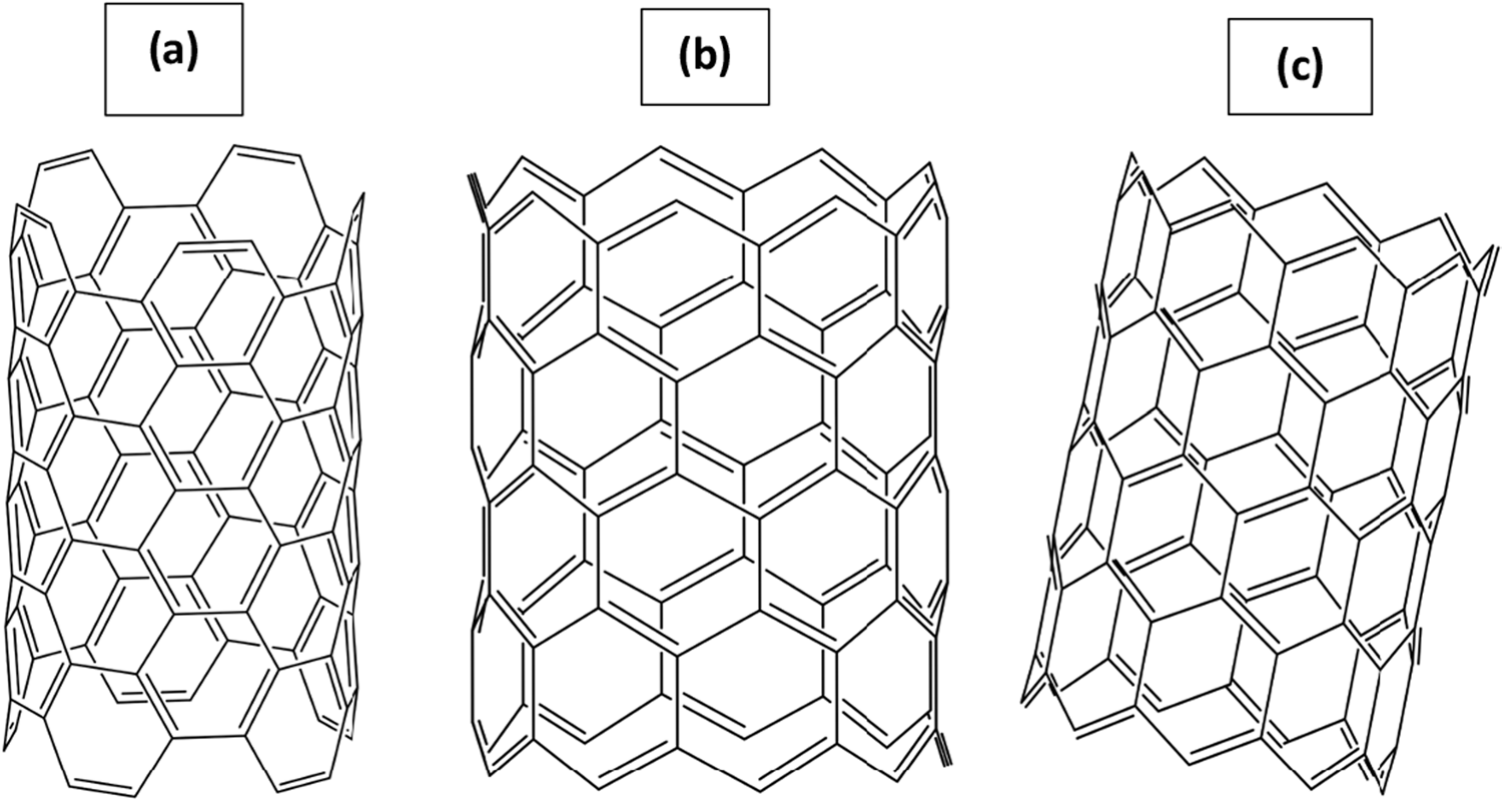
Chirality of carbon nanotubes; Armchair (**a**), ZigZag (**b**), and Chiral (**c**)

Carbon nanotubes, a remarkable class of nanomaterials, exhibit varying lengths and diameters, offering diverse structural arrangements. Depending on their specific structural arrangement, these one-dimensional materials can function as conductors, semiconductors, or insulators. The three primary arrangements observed in carbon nanotubes are chiral, zigzag, and armchair, which are determined by the angle of their structure. The zigzag structure is formed when the angle equals zero, while the armchair structure arises when the angle equals 30 degrees. All other angles give rise to chiral structures, as illustrated in (Fig. 2).

**Fig. 3:**
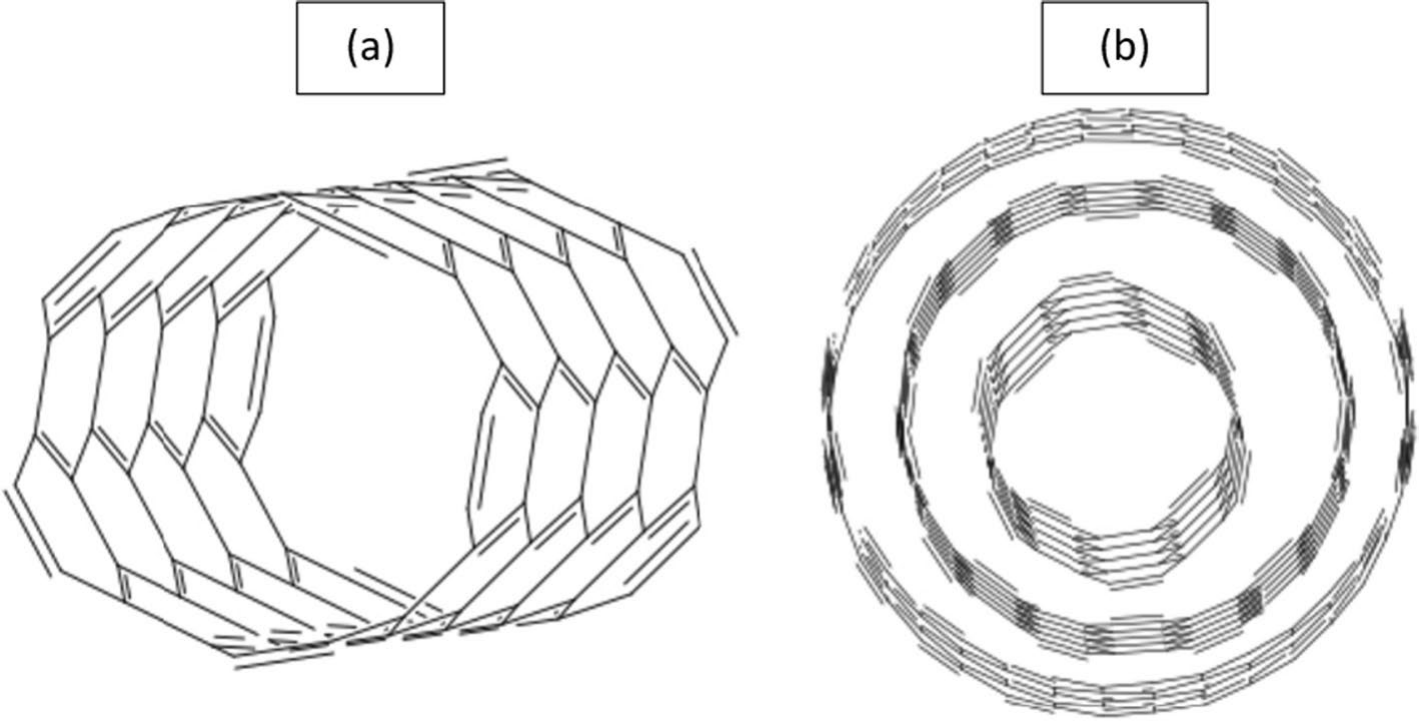
Types of CNTs; single-wall CNT (**a**), and multi-wall CNTs (**b**)

Carbon nanotubes can be categorized into two main types: single-wall nanotubes (SWNTs) and multi-wall nanotubes (MWNTs). SWNTs consist of a single layer of graphite rolled into a cylindrical structure, while MWNTs comprise multiple layers of rolled graphite sheets. This distinction is depicted in (Fig. 3), highlighting the structural variation between the two types.

The manufacturing techniques employed to produce carbon nanotubes encompass several methods, including arc-discharge, laser-ablation, and catalytic growth [13]. These techniques enable the controlled synthesis of carbon nanotubes, allowing for tailored properties and applications in various fields. By comprehending carbon nanotubes' unique properties and manufacturing processes, we unlock the potential for their utilization in a wide range of applications, propelling advancements in nanotechnology and beyond.

Regarding the practical applications of carbon nanotubes, their utilization spans numerous fields owing to their distinctive physical characteristics and chemical stability. These applications encompass energy storage, batteries, electrodes, and the medical domain. In the medical field specifically, carbon nanotubes have found utility in drug delivery. For instance, their extensive surface area enables efficient loading of anti-cancer drugs. Moreover, their heightened permeability allows for enhanced accumulation within solid tumors, surpassing the capabilities of conventional chemotherapy. To illustrate, the conjugation of paclitaxel, an anti-tumor drug, with single-wall carbon nanotubes (SWCNTs) yielded promising results, exhibiting superior tumor growth inhibition while mitigating toxic effects on normal breast tissue.

Nonetheless, poor solubility represents a significant challenge associated with these nanomaterials, highlighting the importance of functionalization. Functionalization serves to enhance both the water solubility and biocompatibility of carbon nanotubes. There are two primary strategies for functionalization: covalent and non-covalent approaches. Covalent functionalization is predominantly employed for drug delivery due to its ease of control and robustness. It is often referred to as defect functionalization since the reaction occurs at the defective carbons at the end or sidewall of the carbon nanotubes. These carbons can be oxidized to generate carboxylic acid groups, subsequently modified through processes such as amidation or esterification. On the other hand, the non-covalent strategy involves either wrapping polymers around the nanotube sidewalls or leveraging p-p stacking interactions between the graphite sheets of the nanotubes and aromatic rings. This technique offers the advantage of preserving the original properties of the carbon nanotubes [14].

Some challenges need to be considered either during the manufacture of CNTs or even before the utilization in general. These challenges start with safety, as the manufactured carbon tubes need to be highly pure to minimize toxic ions that could be released and cause damage to any biological environment. However, to get highly pure CNT, the quality compromises with the quantity, leading to either a high or low production rate. Another challenge is related to the formulation issues, as CNT is hydrophobic, leading to difficulty to solve in water or to be stabilized in suspensions. This issue could be mitigated by functionalizing CNT either covalently (chemical oxidation) or non-covalently (adding surfactants). Moreover, the high viscosity of CNT is also an issue that leads to difficulty in preparing nanocomposite materials [15].

Within the context of this review, which focuses on applying nanomaterials against viruses, carbon nanotubes play a pivotal role as detectors and biosensors for various virus types. Their application as detectors and biosensors is particularly relevant due to their inherent properties.

By exploring the diverse applications of carbon nanotubes and addressing challenges such as solubility through functionalization strategies, we can unlock their full potential in various fields, including medicine and virus detection.

In contrast to traditional methods, biosensors or detectors based on carbon nanotubes (CNTs) offer several advantages, making them superior in terms of time efficiency, cost-effectiveness, and simplicity. These electroanalytical CNT-based sensors possess additional strengths, including the capability to detect substances without interference from excipients endogenous or exogenous compounds. They also exhibit low detection limits of around 10^-7 M or even lower, high selectivity, and the ability to detect redox reactions. This enables insights into drug metabolism and their interactions with proteins.

#### Detection of velpatasvir (VELPR) by MWNTs/AuNPs

In their study, El-Wekil et al. successfully developed a susceptible sensor called a molecularly imprinted sensor (MIS) to detect VELPR directly. They employed a unique approach to enhance the conductivity and surface area of the sensor. They deposited the sensor on an electro-3D starfish-like nickel skeleton, amplifying the signal and increasing its sensitivity. Additionally, they modified the glassy carbon electrode (GCE) using a combination of multi-wall carbon nanotubes (MWCNTs) and gold nanoparticles (AuNPs).

To create a cavity with high affinity and bonding sites specifically designed for VELPR, the electrode was coated with a molecularly imprinted polymer (MIP) using an in-situ electro-polymerization technique. The resulting sensor responded remarkably to VELPR across a wide concentration range of 0.648–80.01 ng mL^−1^. Moreover, it demonstrated exceptional selectivity, sensitivity, stability, and reproducibility. This sensor also proved its capability to determine VELPR concentrations in complex biological systems and tablets accurately [16].

#### Detection of HCV antigen by MultisHRP-DNA-coated CMWNTs

Cuixia Ma et al. reported that the immunosensor detector consists of a modified electrode comprising methylene blue and mesoporous carbon. In addition, a layer of peroxidase-DNA-coated carboxyl multi-wall carbon nanotubes serves as the secondary antibody. The secondary antibody plays a crucial role in providing a wide range of redox enzymes and enabling the detection of signals even at deficient analyte concentrations.

To immobilize the captured antibodies, gold nanoparticles are deposited on the electrode. A complex assembly is achieved by employing the hybridization of biotin-tagged signal and auxiliary probes, along with the bridging probe, secondary antibody, and DNA concatemers. The final step involves utilizing the biotin-streptavidin system to label streptavidin-horseradish peroxidases (HRP) on the antibody, following the findings of Liang et al.

The resulting detector exhibits a high level of selectivity, with an estimated detection limit of 0.01 pg mL^-1^. In summary, the device incorporates GMCs-MB for redox determination, MultisHRP-DNA-CMWNTs as a signal amplifier, and offers a broad dynamic range [17].

#### Detection of daclatasvir (DCV) using Ni-NPs/MWCNTs

As previously mentioned in the DCV/sofosbuvir section, DCV is a direct-acting drug used in combination with sofosbuvir for the treatment of HCV. In their research, Roghayeh Joghani et al. developed a sensor to detect DCV. This sensor utilizes a variety of nickel nanoparticles (Ni-NPs), multi-walled carbon nanotubes (MWCNTs), and a glassy carbon electrode (GCE).

**Fig. 4:**
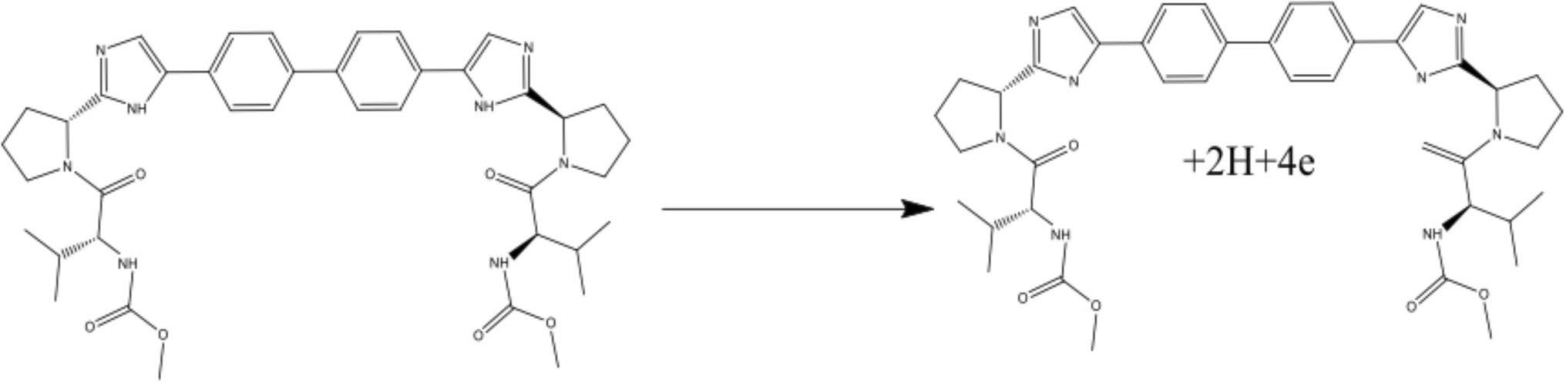
DCV oxidation

The choice of modified metallic nanoparticles (MNPs), specifically nickel, is based on their enhanced properties. These properties include high mass transport, increased surface area, catalytic capabilities, magnetic properties, and biocompatibility. Coating MWCNTs with Ni-NPs achieves a synergetic effect, resulting in an increased active surface area. This, in turn, enhances the oxidation capability of DCV on the sensor's surface, as illustrated in (Fig. 4).

The developed sensor exhibited acceptable and reasonable performance in detecting DCV in real samples, even in the presence of other substances such as uric acid (UA), folic acid (FA), and sofosbuvir [18].

#### Detecting viruses (HCV) by utilizing MWCNTFET

Field effect transistors (FETs) are widely used as bio-detectors because they can quantify charges resulting from biomolecular interactions. In the study by M.F. Fatin et al., a specific FET device was fabricated to detect HIV-1 tat. During HIV invasion, there is an incubation period during which antibodies are still being produced, making it challenging to detect the virus. This can lead to unintentional transmission of the disease. However, the MWCNTFET device can detect HIV-1 Tat by utilizing an aptamer as a probe. Tat, as the first protein produced after complementary deoxyribonucleic acid generation, can penetrate the membrane.

The fabrication process described by Fatin et al. involves several steps. First, the device is created on a silicon wafer, cleaned, and coated with a photoresist layer using a spin coater. The photoresist is then exposed to UV light, and the resulting pattern is checked for defects using a microscope. The top layer of silicon is removed using an etcher and acetone, leaving behind the desired pattern. Nickel and gold metals are deposited onto the patterned device through thermal deposition. Finally, fMWCNT is deposited on the silicon oxide surface channel using a spray method [19].

Alternatively, other devices can be fabricated using the same principle but using single-wall nanotubes (SWNTs) instead of multi-wall nanotubes in the FET. In the study by Dastagir et al., a SWNT-based FET activated with Peptide Nucleic Acid (PNA) is utilized to detect HCV RNA. Chemical vapor deposition (CVD) is employed to deposit aluminum and nickel, unlike the previous device that used gold/nickel deposition through thermal methods. Metallic SWNTs can decrease the sensor's sensitivity, so an electrochemical method is employed to selectively break down the SWNTs, resulting in changes in the resistance between the SWNTs and the metal electrodes.

Overall, both the MWCNTFET and SWNT-based FET devices demonstrate their potential for detecting specific biomolecules and viruses, providing valuable tools for biomedical applications [20].

## Dendrimers

Dendrimers are small spherical molecules with multiple branches, derived from the Greek words "dendron," meaning tree, and "mer," meaning part. They are also known as arboroles or cascade structures. These nanostructures consist of three main components: the core, the outer shell, and the multivalent surface. The multivalent surface contributes to enhanced activity compared to monomeric interactions, known as the dendritic effect or cluster effect, which results in stronger binding to receptors (synergistic effect). Dendrimers exhibit unique chemical and physical properties, such as high solubility, uniformity in size, biocompatibility, low toxicity, and high loading capacity due to their globular structure. It is essential for dendrimers to be non-immunogenic unless used as vaccines and have sufficient circulation time to achieve the desired effect. Also, one main challenge for dendrimers is that by increasing the generation, the toxicity increases [21].

Studies have shown that modifying PAMAM dendrimers, specifically the N-terminated type, with polyethylene glycol (PEG) can decrease immunogenicity and increase their circulation time in the bloodstream. Additionally, dendrimer surfaces can be tailored with T-helper epitopes or antigens to enhance immunogenicity. However, cationic macromolecules, including dendrimers, are generally unfavorable due to their destabilizing effect on cell membranes, leading to increased cytotoxicity and cell lysis. Cell lysis occurs when the positive charge on the dendrimer surface interacts with the negative charge on the cell membrane, causing adhesion and damage to the membrane. Hemolysis and toxicity tend to increase with higher dendrimer generations, particularly for PAMAM and PPI types [22].

**Fig. 5:**
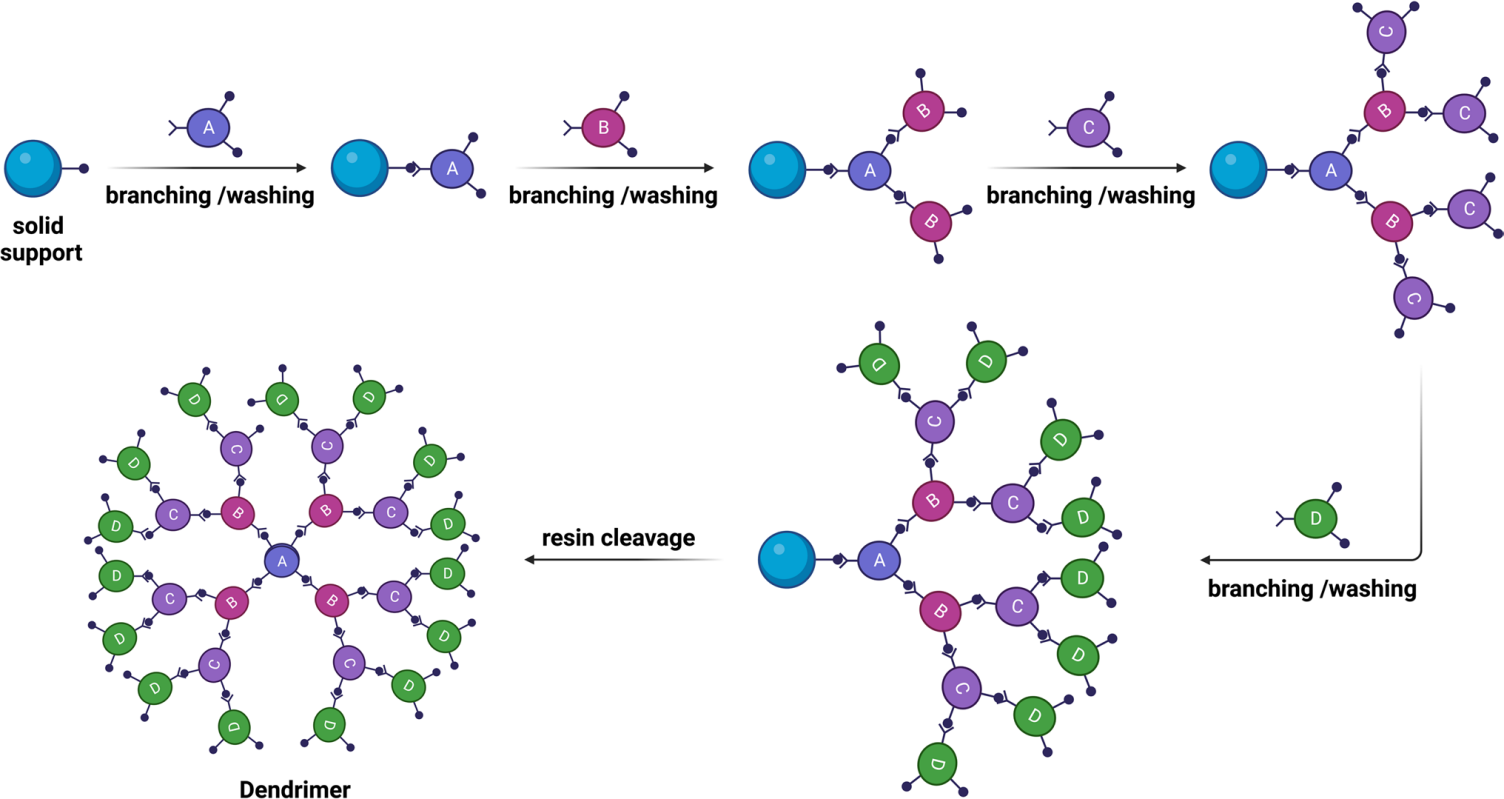
Divergent approach for synthesis of dendrimers. Figure is produced by BioRender.com (2023). Retrieved from https://app.biorender.com/biorender-templates [5]

The structure of dendrimers is characterized by cascades or layers between each core, known as generations. Dendrimers can be classified into various types based on their chemical structure and terminal functional groups, with polypropylene imine (PPI) and polyamidoamine (PAMAM) dendrimers being the most common. Synthesis of dendrimers can be achieved through two methods: the divergent approach, as shown in (Fig. 5), which builds the dendrimer from the core to the branches, and the convergent approach, which synthesizes the dendrimer from the surface to the core. The convergent method allows for the production of asymmetric dendrimeric structures by linking different dendronic segments in a controlled manner.

While dendrimers can mimic certain aspects of proteins, they have significant differences. Dendrimers have covalent bonds within their interiors, resulting in a less flexible and less compact structure compared to proteins of the same molecular weight. Dendrimers also have a higher number of functional groups on their surface. The conformation of dendrimers can be tight (native) or extended (denatured), depending on factors such as polarity, pH, and ionic strength. For example, dendrimers with primary amines on their surfaces, such as PPI and PAMAM dendrimers, exhibit denatured properties at low pH due to electrostatic repulsion between tertiary amines in the interior and primary amines on the surface. At high pH, back-folding occurs due to hydrogen bonding between the tertiary amines in the interior and the primary amines on the surface, leading to a denser core. The charged group nature and pH influence these conformational changes.

Dendrimers find applications in various fields, including scaffolds, antitumor and antibacterial drugs, protein denaturants, vaccines, and antiviral drugs. This review focuses explicitly on the role of dendrimers as antivirals, particularly against HCV (Hepatitis C Virus) [23].

### Polyanionic carbosilane dendrimers (PCDs)

Carbosilane dendrimers possess excellent thermal stability, primarily attributed to the high energy of the C-Si bond. Additionally, their hydrophobic nature can be modified by introducing polar groups through surface functionalization [24]. An example of a carbosilane dendrimer is G2-S24P, which belongs to the second generation and features a polyphenolic core structure accompanied by 24 sulfonate groups immobilized on the surface.

Regarding their mechanism of action against HCV, these carbosilane dendrimers disrupt the early stages of the viral infection process. They achieve this by destabilizing the HCV particles and hindering their attachment to the target cells, effectively blocking viral adsorption. Additionally, macrophages can come into contact with CD4/CD8 cells, resulting in a significantly higher infection transmission rate, estimated to be tenfold. The strategic location and function of macrophages contribute to the continuous spread of the infection. However, the discovery of the G2-S16 dendrimers has shown promising results in combating the spread of disease caused by macrophages. These dendrimers act as antigen-presenting cells for CD4/CD8 lymphocytes while inhibiting viral infection. Specifically, G2-S16 dendrimers function as microbicides, protecting the vaginal mucosa and reducing viral load and reservoirs.

It's important to note that not all polycationic dendrimers are effective against viruses. Dendrimers with a positive charge, such as G2-NN24P and G2-NN16, are either less potent or exhibit cytotoxicity compared to polyanionic dendrimers. However, due to their amphiphilic properties, cationic dendrimers can still be utilized as non-viral carriers for drug and gene therapy in cell transfection protocols. To address the issue of toxicity, the binding of polyethylene glycol (PEG) can significantly decrease the side effect [25].

Studies conducted by Crespo et al. have shown that polycationic dendrimers effectively inhibit both significant genotypes of HCV, including genotype 1a (TNcc) and genotype 2a (JFH-1). Another crucial factor to consider is the timing of administration. Polycationic dendrimers exhibit their inhibitory effect only when applied early in the infection, specifically within the first five hours. They do not have an impact on HCV if used later, indicating that they primarily target the early stages of the viral cycle, particularly extracellular virions, and do not affect post-adsorption processes [26].

### Janus dendrimer (Bis-MPA)

This category of dendrimers utilizes two different dendritic blocks, which can result in synergistic or antagonistic effects. Specifically, Janus dendrimers have been developed by combining hydrophilic and lipophilic dendrons. Moreover, incorporating terminal amino acids into the bis-MPA Janus dendrimer enhances cellular internalization and drug delivery through encapsulation facilitated by forming nano-assembled aggregates in water.

In terms of application, the drug chosen for encapsulation is Camptothecin (CPT), which is primarily used as an anticancer agent but has shown significant efficacy against HCV. CPT acts as an inhibitor of the NS3 protease enzyme involved in the replication process of HCV. However, CPT has certain limitations, including high toxicity, low water solubility, and poor physiological pH stability. Research conducted by Lancelot et al. revealed that Janus dendrimers of the first generation exhibit potent inhibition of HCV replication with minimal impact on cell viability, even at low concentrations of CPT. This outcome demonstrates the superiority of Janus dendrimers over free CPT [27].

## Fullerenes

Fullerenes have versatile and numerous biomedical applications, such as drug-delivery systems, MRI contrast agents, radiation protection, and gene therapy. Focusing on drug delivery, Paclitaxel is a drug approved by the FDA (US Food and Drug Administration) for treating cancer. The fullerene derivative, which is water soluble, enables the uptake of the drug without using a nonaqueous solvent that might cause discomfort for the patient [28]. Two main challenges for fullerenes are low solubility and causing oxidative stress in the brain. According to Zhu et al., fullerenes can cause decreasing glutathione levels and increase oxidative stress in the brain; however, the study was conducted on non-functionalized fullerenes [29].

Exploring new and promising approaches, fullerenes and their derivatives have demonstrated significant potential as antiviral agents due to their unique physical and chemical properties. The motivation behind seeking novel treatments like fullerenes stems from the development of drug-resistant mutant forms of HCV. Notably, research conducted by Mashino et al. highlights that HIV and HCV, both RNA viruses, possess similar enzymes.

Fullerenes have exhibited remarkable efficacy against both HCV and HIV. They synergize by targeting two specific enzymes associated with these viruses. For HIV, these enzymes are protease (HIVP) and reverse transcriptase (HIV-RT) [30]. The impact of fullerenes on HCV and HIV is potent, and their non-toxic nature further enhances their effectiveness in combating HCV infection [31].

### Synthesis of fullerenes

Various techniques are employed to produce fullerenes, including arc-discharge of graphite, combustion, and chemical vapor deposition (CVD) methods. This review primarily focuses on synthesizing fullerenes through the vaporization of a carbon source due to limitations in other routes. The mechanism of carbon source vaporization has been investigated by several scientists, and their contributions will be briefly discussed.

One approach involves laser irradiation of graphite, which results in the production of stable clusters consisting of 60 carbon atoms. However, this method requires extreme conditions, such as temperatures around 1300 °C and pressures of approximately 1 kbar, and the yield is lower than 1%. Additionally, significant effort is required for purification. In an effort to find more commercially viable techniques with higher yields and lower costs, current methods such as pyrolysis, arc-discharge plasma, and radio-frequency plasma are commonly used for the vaporization of graphite.

Once fullerenes are formed, understanding the underlying mechanisms becomes crucial. One of the earliest reports, known as icospiral nucleation, was published by Goroff. According to this report, the formation begins with a C20 molecule with a corannulene-like shape consisting of five hexagons surrounding one pentagon. Two possible formations can occur: nautilus-like spiral shells or quasi-spiral particles. In the nautilus-like structure, the corannulene-like unit grows, eventually forming fullerene structures when the pentagons are correctly deposited. Alternatively, the growth can continue, resulting in the formation of quasi-spiral structures. However, a major discrepancy with this theory lies in the predicted completion time of 10–4 s, whereas experimental observations indicate much shorter timescales, estimated to be between 10–12 and 10–9 s.

Askhabov proposed the concept of "quatarons," intermediate phase clusters that constantly change their forms. It was suggested that liquid quatarons emerge in a supersaturated medium, and the atoms forming quatarons transform into clusters with fixed distances, known as fullerenes. However, this concept has yet to be refuted due to a lack of supporting evidence.

Irle et al. put forth a mechanism called "SHG" (shrinking hot giant), which explains that fullerenes can be formed through two steps: size-up and size-down. In the size-up step, fullerenes are formed from carbon vapor, while in the size-down step, giant fullerenes produce C60 and C70 through the irreversible elimination of carbon molecules [32].

### Amino acid fullerenes derivatives (ADF)

The function of ADF is based on their bivalent metal ions, which enter the hydrophobic regions of proteins in the lipid bilayer of liposomes. By doing so, they alter the activity of enzymes bound to the cell membrane. A notable advantage of this mechanism is its effectiveness against enveloped viruses, as they are not soluble in water. As a result, modified amino acid fullerenes have been developed to effectively inhibit HIV-RT, with the inhibition being enhanced as the positive charges near the C60 cages increase. Additionally, research has demonstrated that the trans-2 isomer of fullerenes has a more significant impact on infected HCV cells, indicating that charges, chirality, and substituents influence antiviral ability. Furthermore, the presence of additional groups in the amino acid type can affect the antiviral effectiveness. For instance, in (Table 2), structures 1 and 2 represent ADF variants. The first structure is more potent due to adding two carboxylic groups to the pyrrolidine ring. In contrast, the second compound is less potent than the carboxylic amino acid fullerene but still more effective than other fullerene derivatives. Despite the insolubility of fullerenes, their structure can be modified by incorporating different groups; however, these modifications vary in their potential [28]. Furthermore, in (Table 2), structure 3 exhibits strong inhibitory activity against NS replication enzyme and protease enzyme while demonstrating low cytotoxicity.

**Table 2:** Derivatives of fullerenes amino-acid

Number	Chemical structure	References
1		[31]
2		[31]
3		[28]

The challenge lies in finding a balance between antiviral effectiveness and low toxicity, which can be achieved by ensuring high solubility. One important aspect is the trade-off between the antiviral activity and cytotoxicity. Additionally, C70 fullerenes have demonstrated antiviral properties, but their synthesis could be more challenging due to issues related to lower symmetry. These fullerenes target the envelope gp120 protein found in HIV and HCV, which serves as an entry point into host cells via specific surface receptors. Now, let's consider non-derivatized fullerenes. Non-derivatized C60 has shown antiviral activity by inhibiting the reverse transcriptase enzyme, although the exact mechanism of action remains unknown [33].

### Conjunction of cyclodextrin (CDs) with fullerene

**Fig. 6:**
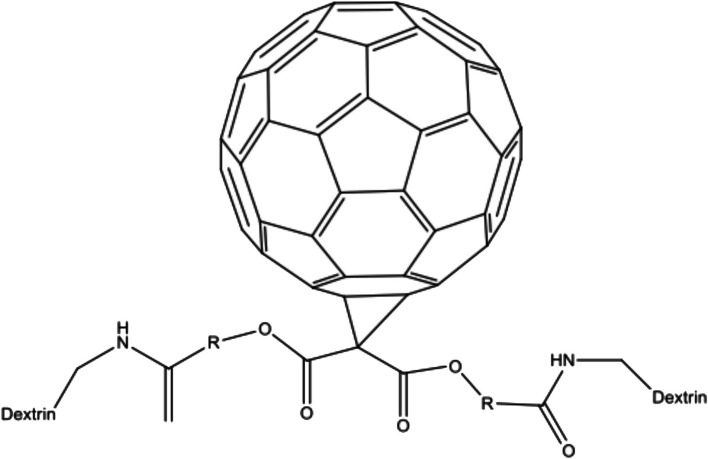
Conjunction of fullerene with dextrin

A significant limitation of C60 is its poor solubility and tendency to form aggregates. To address this problem, complex formations with cyclodextrins (CDs) have been developed, resulting in improved solubility. This complex functions by blocking the entry of HCV through the CD81 receptor. An illustration of this complex can be seen in (Fig. 6), specifically in structure 5, which represents a conjugated CDs-fullerenes complex. According to the study conducted by Xiao et al., this complex demonstrates inhibitory activity during the virus cell fusion process, specifically after virus binding and before virus entry [34].

### Cationic fullerenes

When selecting a fullerene derivative, toxicity and hemolysis are important factors. The presence of a charge plays a crucial role in determining the compound's toxicity. For instance, compounds 1 and 2 in (Table 3) exhibit high toxicity due to their cationic charge. These compounds possess an amphoteric property that disrupts the cell membrane, resulting in a toxic effect. In contrast, compounds 3 and 4 in (Table 3) do not demonstrate cytotoxicity or hemolytic effects, which can be attributed to the presence of carboxylic groups. There might be a relationship between the hemolytic activity and the total hydrophobic surface area (ASA_H) compared to the total hydrophilic surface area (ASA_P).

**Table 3:** Fullerene derivatives structures from 1 to 5

Number	Chemical structure	References
1	(trans-2)	[35]
2		[34]
3		[34]
4		[34]
5		[35]

To provide further clarification, referring to (Table 4), structure 1 corresponds to a proline-type fullerene derivative, while structure 2 represents a pyridinium-type derivative. The proline-type derivative exhibits inhibitory effects on HCV NS3/4A and HCV NS5B, highlighting the dual impact of fullerenes in combating HCV. When considering pyridinium/carboxylic acid-type derivatives, both cis-4e and cis/trans-4f demonstrated higher effectiveness and potency as HIV-PR inhibitors than cis-3a. However, cis-4a was found to be less effective in this regard. On the other hand, dicationic fullerenes such as cis/trans-3i, cis-3k, and cis-0–31 exhibited greater potency than other types, particularly in inhibiting HCV NS5B. The inhibition efficacy in relation to HCV NS5B primarily depends on the fullerene backbone [36].

**Table 4:** Chemical structure of compounds fullerene derivatives

Number	Chemical Structure	References
1		[37]
2		[37]
2	2a	[37]
3	2b	[37]
4	Trans-3a,Cis-3a	[37]
5	3b	[37]

Another novel fullerenes compounds, presented in (Table 4), the proline type derivatives, 2a and 2b, are more potent than cis-3a and cis-3b, pyridinium type; however, the mentioned pyridinium derivatives are more potent than structure number 1 (pyridinium type). Moreover, structure 2a, number 2, exhibited a dual inhibitory, multi-target, effect by its effect on both HCV NS3/4A protease and NS5B polymerase.

## Challenges and future recommendations

Carbon allotropes are a promising upcoming field of study due to the features that these unique nano structures provide compared to the conventional treatments; however, many challenges need solutions or at least mitigations to say that we, as researchers and scientists developed better medications. To justify, starting with CNTs, most CNT applications mentioned in this review and have been used up to this date against HCV are sensors only used for identifying the drug concentration administrated to treat HCV. Based on this fact, what about identifying the virus infection itself early, and which genotype to interfere more effectively. In essence, developing more effective sensors for genotype classification and determination is needed because some medications work on specific genotypes while others need to be fixed. Moving to dendrimers, not all dendrimers have an effect against HCV, especially the cationic and polycationic types. However, some newly discovered dendrimer types, such as Janus and anionic dendrimers, have promising effects. This leads to the importance and the need for more investigations, as anionic dendrimers are unique due to the C-Si bond, there might be more potent types. Although fullerenes showed an effective mechanism against HCV, they are highly insoluble, which addresses the need to find fullerenes derivatives that can mitigate this issue. This is one of many issues in fullerenes, as their synthesis cost is extremely high, and the yield is 1% or less. To overcome this, the preparation of fullerenes and altering their properties should be done by using computational and simulation studies. Doing this will decrease the cost significantly, save time, and prepare only the desired structure along with their properties. Moreover, I believe the future of drug advancements relies in this area because nanoscience is still under development, and more is yet to be discovered. and even with the low knowledge that we knew and have discovered till today, it is very promising compared with the traditional and old treatments. To emphasize, (Table 5) show the commercialization and market value and the increased demand, especially for multi-walled carbon nanotubes, which increased by approximately fourfold, from 205.1 million dollars in 2012 to 813.4 in 2022.

**Table 5:** Market value for CNTs worldwide

	2012 (million U.S dollars)	2020 (million U.S dollars)	2022 (million U.S dollars)	References
Single-walled carbon nanotubes	21.4	46.6	64.5	[38]
Multi-walled carbon nanotubes	205.1	618.1	813.4	[38]

**Fig. 7:**
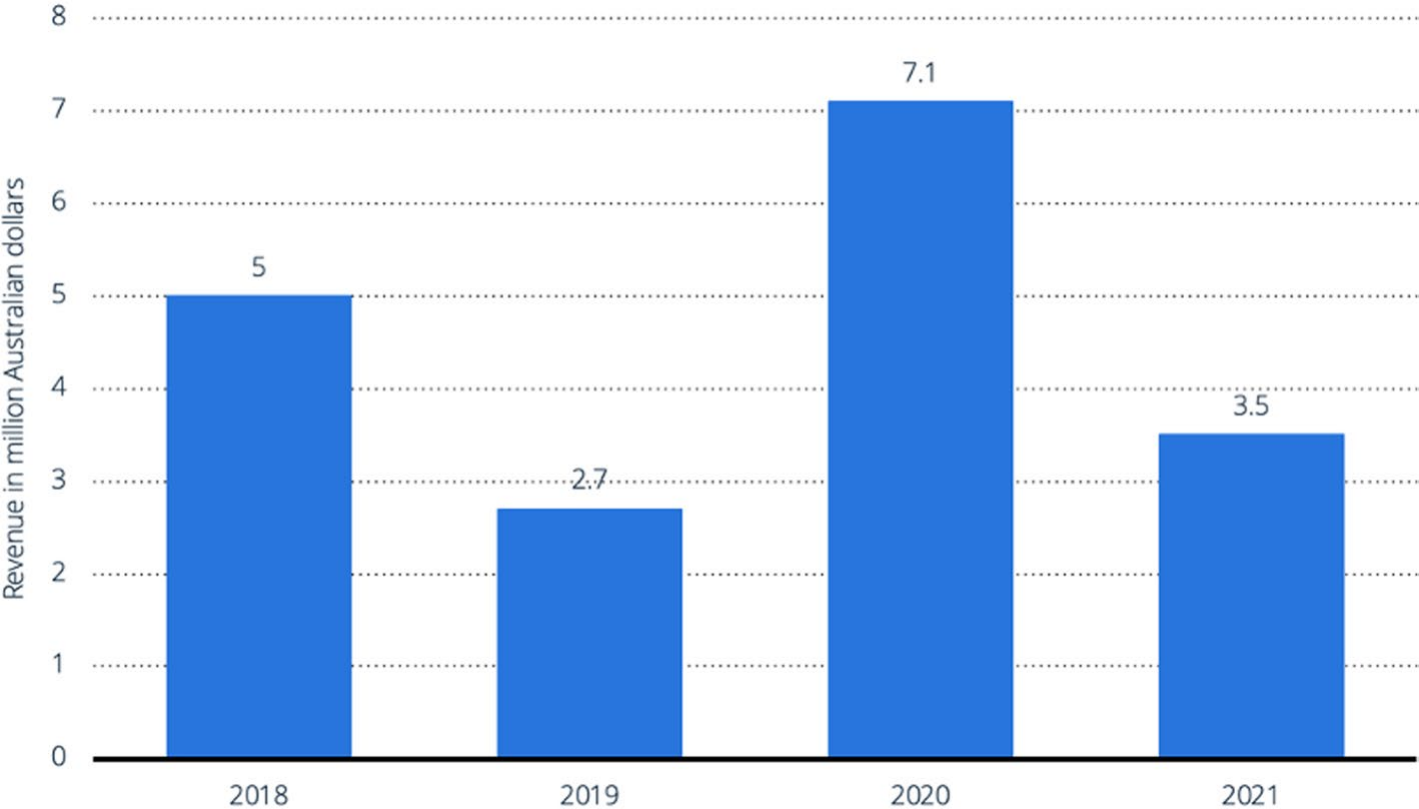
Starpharma Revenue from dendrimers production [39]. Figure is retrieved from Statista (2023)

There is no available data for fullerenes; the reason may be due to the high production cost and low yield, which agrees with what has been discussed above. Regarding dendrimers, the data that has been found so far is for StarPharma in Australia, shown in (Fig. 7) representing the increase in revenues over the period of the four years from 2018 to 2021[39].

## Conclusion

Developing new and innovative drugs is crucial to overcome and prevent the emergence of viral mutant resistance genes against current treatment protocols. It is also necessary to discover more potent treatment options. The treatment began with IFN, which was less potent and had more side effects than the more powerful combination therapy involving sofosbuvir. However, these combinations cannot be administered as monotherapy to prevent the development of viral tolerance and resistance. Additionally, each combination therapy is specific to certain virus genotypes, necessitating the identification of the infecting species before initiating treatment. Nanotechnology has opened up new possibilities, offering versatile structures that can be more effective and less toxic. Numerous ongoing research investigations utilize nanostructures' unique features for drug delivery against HCV and HIV. Amino acid fullerenes have emerged as promising compounds in the quest to eliminate HCV, as they have demonstrated dual inhibitory effects compared to other treatment protocols that only target a single mechanism. However, certain mechanisms of action still need to be fully understood and require further investigation. It is important to note that an HCV vaccine is not yet available. Carbon nanotubes, with their versatile structures, are utilized as detectors due to their high surface area, which enables high sensitivity. They can be employed for treatment or antigen detection purposes. Furthermore, various types of dendrimers are used for drug encapsulation to minimize toxicity or enhance drug delivery and absorption. Lastly, it is important to highlight that there is currently no treatment available that is effective against and eradicates all genotypes of these viruses.

## Data Availability

All data underlying the results are available as part of the article and no additional source data are required.
